# Art psychotherapy meets creative AI: an integrative review positioning the role of creative AI in art therapy process

**DOI:** 10.3389/fpsyg.2025.1548396

**Published:** 2025-03-20

**Authors:** Ania Zubala, Alison Pease, Kacper Lyszkiewicz, Simon Hackett

**Affiliations:** ^1^Centre for Clinical Brain Sciences, College of Medicine and Veterinary Medicine, University of Edinburgh, Edinburgh, United Kingdom; ^2^Department of Computing, School of Science and Engineering, University of Dundee, Dundee, United Kingdom; ^3^Independent Researcher, Edinburgh, United Kingdom; ^4^Population Health Sciences Institute, Faculty of Medical Sciences, Newcastle University, Newcastle upon Tyne, United Kingdom; ^5^Cumbria, Northumberland, Tyne and Wear NHS Foundation Trust, Newcastle upon Tyne, United Kingdom

**Keywords:** art therapy, art psychotherapy, artificial intelligence, generative AI, creative AI, mental health, digital health, integrative review

## Abstract

**Background:**

The rise of artificial intelligence (AI) is promising novel contributions to treatment and prevention of mental ill health. While research on the use of conversational and embodied AI in psychotherapy practice is developing rapidly, it leaves gaps in understanding of the impact that creative AI might have on art psychotherapy practice specifically. A constructive dialogue between the disciplines of creative AI and art psychotherapy is needed, to establish potential relevance of AI-bases technologies to therapeutic practice involving artmaking and creative self-expression.

**Methods:**

This integrative review set out to explore whether and how creative AI could enhance the practice of art psychotherapy and other psychological interventions utilizing visual communication and/or artmaking. A transdisciplinary search strategy was developed to capture the latest research across diverse methodologies and stages of development, including reviews, opinion papers, prototype development and empirical research studies.

**Findings:**

Of over 550 records screened, 10 papers were included in this review. Their key characteristics are mapped out on a matrix of stakeholder groups involved, elements of interventions belonging to art therapy domain, and the types of AI-based technologies involved. Themes of key significance for AT practice are discussed, including cultural adaptability, inclusivity and accessibility, impact on creativity and self-expression, and unpredictability and imperfection. A positioning diagram is proposed to describe the role of AI in AT. AI’s role in the therapy process oscillates on a spectrum from being a partner in the co-creative process to taking the role of a curator of personalized visuals with therapeutic intent. Another dimension indicates the level of autonomy – from a supportive tool to an autonomous agent. Examples for each of these situations are identified in the reviewed literature.

**Conclusion:**

While creative AI brings opportunities for new modes of self-expression and extended reach of art therapy, over-reliance on it presents risks to the therapy process, including of loss of agency for clients and therapists. Implications of AI-based technology on therapeutic relationship in psychotherapy demand further investigation, as do its cultural and psychological impacts, before the relevance of creative AI to art therapy practice can be confirmed.

## 1 Introduction

The rise of artificial intelligence (AI) is having an increasing and at times profound impact on all areas of life, including health and medicine. Large data processing capabilities and the ability to detect complex patterns by powerful algorithms have already guaranteed a long-standing place for AI in biosciences, diagnosis and treatment of physical health conditions, notwithstanding ongoing discussions on ethical, social and cultural implications of welcoming AI to such a vulnerable and personal space of human wellbeing. A place for AI within the mental health domain is arguably even less straightforward, raising fundamental questions on the future of human-specific (until now at least) psychological phenomena like emotional regulation, relationship building and maintenance, ability to reflect, self-consciousness, and many more.

### 1.1 Application of AI in psychotherapy

In psychotherapy, any potential application of AI within therapy sessions is highly debatable (e.g., Fiske et al., 2019; Haber et al., 2024; Sedlakova and Trachsel, 2023), primarily due to the fundamental concept of the therapeutic relationship traditionally intuitively understood as human-to-human exclusively. Introduction of AI in psychotherapy does not necessarily mean replacement or even reduction of this central therapeutic factor. However, we should expect AI’s presence in the therapy space to affect the dynamic of the therapeutic relationship and inevitably impact the therapy process and its curative potential – in ways difficult to predict.

Insightful discussions on the implications of introducing AI in (talking) psychotherapy are underway, focusing on embodied AI – that interacts with its physical environment (Fiske et al., 2019), and conversational AI – that engages in dialogue (Grodniewicz and Hohol, 2023; Miner et al., 2019; Sedlakova and Trachsel, 2023), as naturally relevant to therapy processes developing primarily on the basis of verbal communication and embodied presence. There is no unequivocal agreement as to the extent of the impact that AI would have on psychotherapy practice, with descriptions of its potential role ranging from an “add-on resource” (de Mello and de Souza, 2019), through a “nuanced technological tool” (Haber et al., 2024), to “a new artifact that can change our interactions, concepts, epistemic field, and normative requirements” (Sedlakova and Trachsel, 2023). Psychotherapy scholars generally seem to agree that AI, in its current state of development at least, would not be able to mimic the entire complexity of therapist-client interaction nor lead to a depth of insights or therapeutic change achievable only via human-human therapeutic relationship (Grodniewicz and Hohol, 2023; de Mello and de Souza, 2019; Sedlakova and Trachsel, 2023). However, while present days AI might inevitably have a limited role to play within the currently acceptable psychotherapy paradigms, most authors seem to sense that it nevertheless cannot be ignored as potentially transformative force, likely to create completely novel scenarios in which to practice psychotherapy (Grodniewicz and Hohol, 2023; Sedlakova and Trachsel, 2023; Fiske et al., 2019). Indications of great opportunities are balanced in the literature with cautionary hints of equally great dangers of introducing AI in psychotherapy and the wider mental health treatment domain (Haber et al., 2024), where neither suitable ethical guidelines nor training for health care professionals are available (Sedlakova and Trachsel, 2023) to support safe, meaningful and human-centered development of AI-mediated therapy interventions.

### 1.2 Defining art therapy and creative AI

While the above indications from the wider psychotherapy area are highly relevant to potential introduction of AI to art therapy practice, the discipline might be in a unique position to utilize technological features beyond conversational and embodied AI, often referred to as creative AI. Before we go any further, it feels important to clarify what we mean by art therapy and creative AI, particularly as both terms are commonly present in the wider public awareness but frequently used to describe very different concepts and practices.

Art therapy is a form of psychotherapy with a strong evidence base in the treatment of a range of mental health conditions, including depression and anxiety (e.g., Uttley et al., 2015; Blomdahl et al., 2013; Abbing et al., 2018). In art psychotherapy, creative self-expression (typically via artmaking) within a safe therapeutic relationship is used to reduce psychological distress, improve mental wellbeing, and promote insight leading to personal growth. Trained and registered art therapists are health professionals who help their clients address a range of psychological issues that might be difficult, distressing, and potentially hard to verbalize. British Association of Art Therapists highlights that artmaking within art therapy context is a means to “express and articulate often complex thoughts and feelings” (British Association of Art Therapists, 2024a), while the American Art Therapy Association additionally emphasizes the supportive role of art therapists in helping clients “integrate nonverbal cues and metaphors that are often expressed through the creative process” (American Art Therapy Association, 2024). Art therapy practice is based on fundamental principles and ethical guidelines, including, for example, duty of care toward clients and a requirement for regular supervision (e.g., British Association of Art Therapists, 2024b). Art psychotherapy interventions are increasingly manualised and evidenced to best address the needs of specific populations and settings (e.g., Watts et al., 2024; McDonald et al., 2024; Zubala et al., 2023). Terms “art therapy” and “art psychotherapy” are protected titles of a registered profession in the UK, regulated by Health and Care Professions Council (2024), and are subject to credentialling and testing processes in the US prior to registration (American Art Therapy Association, 2022). Both “art therapy” and “art psychotherapy” are commonly used interchangeably and an acronym of “AT” will also be used throughout this paper.

Creative artificial intelligence (AI) can be understood as a subfield of AI which combines technical advances with knowledge from psychology and related fields on creativity, and methodologies from computer science, to simulate creative processes on computers. Advances in the field are driven by a vision to both enhance human creativity and develop autonomous stand-alone creative systems, with arguments being put forward that AI holds the potential to complement rather than simply replicate human creativity (Garcia, 2024). We will use the term “creative AI” throughout this paper for simplicity, recognizing that alternative terms have been argued to more adequately represent the same concept, including “computational creativity” (Colton and Wiggins, 2012; DiPaola et al., 2018) and “artificial creativity” (Runco, 2023a).

### 1.3 Connecting art therapy and creative AI

Powerful AI techniques such as deep learning, and greater ease of use in deploying generative systems have led to widespread innovations in the arts and sciences. The release of large language models (LLMs) such as ChatGPT and the latest generation of generative AI artmaking systems, such as Stable Diffusion, Midjourney and DALL-E, has accelerated the debate on the responsible use of AI, raising questions about copyright, artist empowerment, training biases, diversity, and data security, among others. Research in creative AI is predicted to be developing fast (Mejia et al., 2021). However, to further develop in a healthy and ethical way, the discipline needs to engage with practitioners from diverse fields to cultivate new understanding, suggest meaningful directions, and develop responsible and desirable ways in which creative AI can enhance human lives and societies. Potential enhancement of (psycho)therapeutic practice with AI demands a particularly close collaboration between computer scientists, developers and psychotherapists (Haber et al., 2024), as well as patients, their families and the wider public (Carr, 2020; Zidaru et al., 2021).

Due to the unique and central focus on creativity and artmaking in art psychotherapy, technologies which assist users in making art have been naturally and gradually integrated in practice, playing an important role in the therapy process. These range from physical arts media of all sorts to, more recently, digital image making software, and even virtual reality environments (Hadjipanayi et al., 2023; Hacmun et al., 2018; Orr, 2010). The last couple of decades have seen an increasing interest and ongoing discussion on the application of digital technology to art therapy practice, potentially beyond the use of digital artmaking tools (Zubala et al., 2021), and art therapists have long been open to the idea of collaborating with technology designers (Gussak and Nyce, 1999). However, early attempts to develop bespoke artmaking software/apps for use in art therapy (Choe, 2014; Mattson, 2015) failed to have a transformative effect on practice due to technological limitations in human-computer interactivity and the machine’s limitation in mirroring the creative process, ultimately posing reservations on their relevance to therapy.

The recent dramatic advancements in AI promise completely novel opportunities, not yet explored, including for computationally creative systems to play a significant role in the therapy process and relationship. As such, creative AI has the unique potential to be in fact more relevant to practice than previous (digital) technologies, and possibly transformational in terms of benefits for art therapy clients. At the same time, introduction of AI in art therapy space presents unique challenges, demanding careful consideration from therapists and the wider mental health community. While the risks and benefits of introducing earlier digital technologies in art psychotherapy practice have been widely explored (e.g., Peterson, 2010; Kapitan, 2009; Zubala and Hackett, 2020), potential therapeutic applications of creative AI demand a dedicated investigation of impacts, including on the therapy process, self-understanding and identity of both therapists and clients.

In several years leading to the current work, we have independently performed preliminary explorations into the use of digitally creative systems in therapeutic contexts (Cheatley et al., 2019; Pease et al., 2022; Cheatley et al., 2022; Zubala and Hackett, 2020; Zubala et al., 2021). These efforts highlighted to us both the promise and the necessity of bringing creative AI and art psychotherapy together for a constructive dialogue and potential advancements in both fields.

### 1.4 Aims

The current review is the first step toward evaluating and realizing the potential and relevance of creative AI to art therapy practice. Based on our previous experience with related research on the use of digital technology in AT more generally (Zubala et al., 2021), we expected to be highlighting safety and ethical concerns, any accounts of patient/client and professional experiences of working with AI therapeutically, and evidence of successful implementation (if available).

Our main aim was to explore whether and how creative AI can enhance the practice of art psychotherapy and closely related psychotherapeutic interventions utilizing visual communication and/or artmaking (understood here as within-session work with clients).

More specifically, we searched for answers to the following research questions:

- What are the key challenges and benefits in introducing creative AI to art psychotherapy practice?
- How can creative AI be safely, meaningfully and successfully applied to art psychotherapy practice?
- How can creative AI researchers and art therapists work together to combine and extend the strengths of each discipline, for the benefit of people with experience of mental ill health?

## 2 Methodology

An integrative review format (Whittemore and Knafl, 2005) seemed most suitable for bringing together the rapidly developing research, with its heterogeneity in terms of research designs and quality, as well as the presence of important and influential but non-empirical opinion papers. We took a semi-systematic approach to devising a search strategy that would identify any relevant literature making links between AI, mental health, and psychotherapy practice utilizing artmaking, including published papers and reports and unpublished sources (such as high-quality dissertations and expert opinions, including from people with lived experience of mental ill health).

As an important part of the review process, we purposefully selected a small patient and public involvement (PPI) group of four people familiar with mental health (either via own lived experience or professional practice) and AI (either via practice/training or personal interest). We consulted the PPI group on our search strategy and asked for guidance on areas of priority for our investigation and future research. The PPI group were invited to contribute to the review process through a shared online whiteboard, individual consultations and two focus groups. The latter contributed greatly to identifying themes of key significance to AI-mediated AT practice (see section “Findings”).

### 2.1 Search strategy

Having conducted several “mock” searches, we settled for the following search string: [(“art *therap*” OR “creative *therap*”) AND (“artificial intelligence” OR AI)]. We initially experimented with more complex searches, including, among others, terms such as “generative model*,” “algorithm*,” “human-computer” and “digital co-creat*.” However, although more detailed search strings generated more records overall, they did not result in more records relevant to this review. A simplified search string seemed to capture relevant literature more precisely.

We conducted searches in the key health and computing sciences databases (APA PsycNet, CINAHL, MEDLINE, PubMed, ACM, Scopus, Web of Science, IEEE Xplore), as well as via PROSPERO and Cochrane registers of systematic reviews, and in journals specific to disciplines of arts psychotherapies and creative AI (i.e., The Arts in Psychotherapy, International Journal of Art Therapy, Journal of Creativity in Mental Health, Journal of Medical Internet Research, Frontiers in Psychology, The Journal of Computational Creativity, Digital Creativity, International Journal of Design Creativity and Innovation). We also searched for in-progress studies and initiatives through our respective professional communities and, as far as possible, scanned unpublished literature, conference proceedings and dissertations (e.g., using ProQuest and conference programs). We supplemented our database searches with Google Scholar searches, screening first 300 records for [“art therapy” and “AI”] and 60 first records for [“computational creativity” and “art therapy”] (The latter search was added once it became apparent that the initial search via Google Scholar did not capture some of the literature we knew existed).

### 2.2 Inclusion/exclusion criteria

Any type of literature was considered for this review, including research papers, opinion pieces, reports, conference proceedings, book chapters, dissertations, preprints, etc. Any type of methodology (or no formal methodology) was considered, including empirical studies and opinions, reviews, vignettes, practice papers, etc. We anticipated that texts published before 2014 (10 years prior to this review) were unlikely to concern AI in its most current forms and therefore might be outdated for the purpose of this review (however, time of publication was not an exclusion criterion). A pragmatic decision was made to focus only on the literature available electronically online. However, in contrast to the usual practice of reviewing in an English-speaking context, we decided not to exclude texts based on the language they were published in, provided they were captured by our search string (e.g., abstract available in English).

The key inclusion criteria oscillated around the relevance of content and its potential value for art psychotherapy practice, which in this review we understand to be psychotherapeutic practice aiming to increase psychological wellbeing or improve mental health through visual creative self-expression within a safe therapeutic environment and a supportive therapeutic relationship.

Articles were included in this review if they:

- Focused on the application of artificial intelligence (AI) to art psychotherapy (AT) practice
- Considered psychological/wellbeing outcomes and/or insights directly related to mental health
- Focused on within-session use of AI in AT, i.e., AI present within therapy space, either physical or virtual, and/or within therapist-client therapeutic relationship

Articles were excluded if they:

- Focused on the use of AI within other therapeutic traditions/approaches, such as (verbal) psychotherapy and other arts therapies modalities (music, drama, etc.)
- Focused on the use of AI in assessment, training, admin, or for diagnosis purposes exclusively
- Pertained to novel technologies with no AI systems involved (e.g., virtual reality, extended reality, digital artmaking)
- Focused on the use of AI in areas of creativity or emotions/wellbeing but without explicit therapeutic intent
- Were considered of a quality insufficient for at least low-moderate confidence in the findings/claims expressed

**Figure 1:**
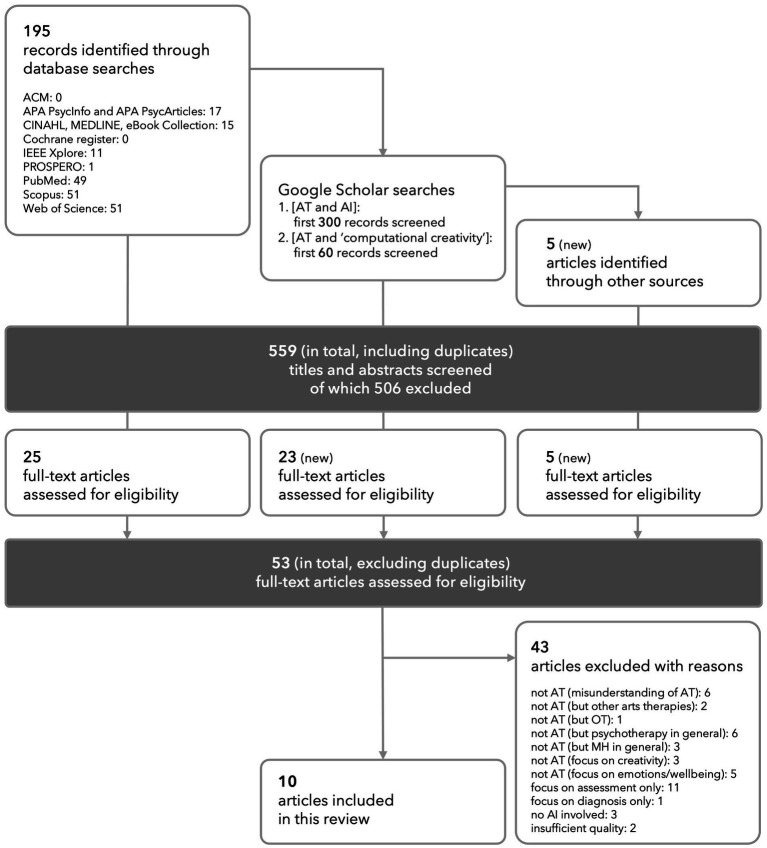
PRISMA flow diagram.

While our search strategy was intentionally designed as a pragmatic and agile system to identify literature from multiple disciplines, methodologically and philosophically largely varied, we followed the PRISMA process (Moher et al., 2009) while searching for and selecting articles (Figure 1) for a transparent documentation of the steps involved.

### 2.3 Quality assessment

Unable to identify any single assessment tool capable of consistently evaluating texts of any type or studies of mixed methodology, we chose to undertake a pragmatic simplified quality assessment of papers included, focusing on the confidence in findings or claims made by authors and the overall credibility of the publication. We based our quality scores on: evidence of the peer review process, clarity of arguments brought forwards, transparency of reporting (including sufficient details on methodology, if applicable), overall quality of academic writing, and demonstrated sufficient understanding of the nature of both disciplines of interest. While non-peer reviewed publications (i.e., conference proceedings) were included in our review, we reflected the lack of the peer review process in lower scores on confidence in their findings. Quality assessment was undertaken by two authors (AZ and KL) and justification for scores is included in Table 1.

**Table 1:** Characteristics of included papers, conceptualisation of art therapy (AI) and creative artificial intelligence (AI), and indicated role of creative AI in AT.

Author(s)/year/country	Type of publication	Aim	Methodology (M) /intended population (IP)/participants (P)	Confidence in findings/opinions	How is AT described?	How is creative AI described?	Indicated role of creative AI in AT
(1) AlSadoun et al. (2020) Saudi Arabia	Conference proceedings: 22nd International Conference on Human-Computer Interaction (HCI International 2020), Copenhagen, Denmark	“To build a smart art therapy system that autonomously communicates instructions and information to users independently from human guidance”	M: prototype development planning (theoretical rationale) IP: people with complex communication needs (autism spectrum disorder, learning or developmental disabilities)	Low-medium: peer-review process unclear, literature review on AT sound and relevant to practice in SA, very early prototype development work with limited details of the actual system	- AT in the context of Saudi Arabia specifically - role of art therapist not clear (system designed to work autonomously)	- Digital artmaking software with an AI component that adjusts the content based on users’ emotions and “an embodied agent that responds to users independently of any expert human guidance” - “Virtual embodied agent plays the role of an art therapy assistant by providing detailed directions for users to follow as they work while still encouraging abstract and imaginative thinking”	- “Increasing the user engagement and satisfaction” - facilitating “comfortable, creative, and therapeutic zones for people with communication difficulties”
(2) Bossema et al. (2023) Netherlands	Conference proceedings: 32nd IEEE International Conference on Robot and Human Interactive Communication (RO-MAN 2023), Busan, Korea	To “explore how social robotics and AI-generated content can support creative experiences of older adults”	M: scoping review IP: older adults	Medium-high: peer-review process unclear, sound methodology, demonstrated understanding of CC and therapeutic application of AI	- AT as one of the discussed applications of HRCC / social robots - Importance of a robot to “understand and adapt to the creative and emotional expressions of a human interaction partner” - “Verbal and vocal” channels of communication highlighted	- Focus on HRCC (Human-Robot Co-Creativity) and Computational Creativity - Social robots: “offer unique opportunities for embodied interaction, sharing agency, and (non-)verbal communication” (specific focus on collaborative drawing and painting)	- Supporting AT through “responsiveness and personalization” (e.g., responding to human creative expression, expressing matching emotions, suggesting visual metaphors) - “Recognizing, modeling, and synthesizing emotions in drawings and paintings” - Robot speech can be used to “demonstrate verbal creativity, scaffold creativity, and promote creative reflection”
(3) Choe and Hinz (2024) USA	Journal article: Art Therapy	To “review definitions of creativity in the age of AI, explore the role and significance of human creativity using the framework of ETC, and imagine effective ways to augment human creativity with AI tools”	M: review of literature, case studies/ vignettes	High: peer-reviewed publication, high quality academic writing, in-depth knowledge of AT discipline and the wider MH context	- AT within the ETC (Expressive Therapies Continuum) model that “can provide a framework that delineates human creativity, from process to product, considering various contextual, developmental, and situational factors”	- Text-to-image generative AI tools that produce visual images (e.g., Midjourney, DALL-E)	- “Generative AI tools can be instrumental in art therapy to efficiently produce positive images of repariation, reminiscence, closure, hope, and more” - “An assistive tool to help clients creatively re-envision and explore various narratives” - “Generative AI has the potential to democratize artmaking for individuals across a spectrum of abilities and skills”
(4) Cooney and Menezes (2018) Sweden	Journal article: Multimodal Technologies and Interaction (MDPI)	To “review literature on robots used for therapy and art, potential strategies for interacting, and mechanisms for expressing emotions and creativity”; to “propose a design for an art therapy robot”	M: early prototyping (“a basic design for an art therapy robot”)	Medium: peer-reviewed publication, no specific methodology, elements of narrative literature review, detailed justification for a robot prototype	- AT defined as “a therapeutic process involving art-making: a patient expresses emotions through creating art, which also serves as a bridge between the patient and a therapist” - “There is no one ‘accepted’ way to conduct art therapy”	- “An autonomous robot capable of painting with a person,” responding to emotions and able to “convey appropriate emotions in a creative manner” - “Affective computing” and “artificial creativity” paradigms - Baxter robot: a humanoid of adult size, with a face display showing various expressions	- To support wellbeing “by engaging with people on an emotional and creative level” (displaying matching or complementary emotions - verbally or via artmaking) - Potential for saving time for human therapists, “leveraging abilities not normally available to humans, such as inferring emotions from brainwaves,” being available at any time - “Facilitating self-exploration without requiring people to express vulnerable thoughts to another human”
(5) Du et al. (2024) Canada / China	Journal article: International Journal of Human-Computer Studies	To “investigate the potential of introducing a human-AI co-creative process [via DeepThInk system] into art therapy”; to “understand how AI could be introduced as a material in art therapy and investigate its meaning based on ETC”	Prototype development via “iterative and longitudinal design process” M1: expert reviews, interviews P1: 5 registered art therapists M2: mock therapy sessions, user testing, interviews P2: 10 volunteers (18–30)	High: peer-reviewed publication, sound methodology, good academic writing	- Focus on “digital art therapy” - AT within the ETC framework and based on AAAT definition (i.e., therapist’s role, verbal/non-verbal communication, non-interpretation) - “The inspirations from DeepThInk depend on participants’ own interpretations, while the art therapist could provide guidance and suggestions which help them see things from a new perspective”	- “A novel AI-infused digital art-making system, DeepThInk” - designed specifically for AT (allowing users to make artworks collaboratively with AI) - “AI techniques should be seen as art materials, rather than automators of the art-creating process” - Human-AI co-creative approach “as a novel yet meaningful form of digital art therapy”	- AI-generative function can simplify the drawing process (“making drawing process more effortless could potentially reduce the initial frustration and hesitation about the art creation, and encourage more participation, self-expression and self-exploration”) - Supporting expressivity, creativity and engagement in AT - The unpredictability of AI “creates space for exploration and creativity”
(6) Harwood et al. (2019) UK	Journal article: OBM Geriatrics	To review literature on the therapeutic use of AI in dementia care	M: narrative literature review IP: people living with dementia	Medium: peer-reviewed publication, non-systematic review	- AT not discussed in detail, focus on “arts at a medium for care and self-care in dementia” - AT recognized as improving quality of life and general cognitive functioning for people with dementia	- AI seen as a tool, “added to the growing list of art-making techniques” - Focus on “the exposure to an algorithm-created digital art”(with “potential to be customized in real-time, reflecting the hyper-personal needs and interests of people living with dementia”)	- AI can “facilitate a person with dementia to be an integral figure in the process of interactive appreciation [of art]” - Potential for “stimulating senses, improving communication and overall enjoyment” - Due to their “psychadelic and fantasmorganic” properties, “imaginary unreal art presentations are not only well received by people with dementia, but cause more curiosity and interactions than the traditional forms of art”
(7) Kwon et al. (2024) Republic of Korea	Conference proceedings: 6th International Conference on Artificial Intelligence in Information and Communication (ICAIIC 2024), Osaka, Japan	“To experimentally ascertain whether the creation of visual images through generative AI leads to psychological improvement for creators or viewers”	M: pre-post study (pre-post survey of “psychological states” and evaluation of co-created images on mimesis, imperfection, catharsis, quality) P: 40 middle-aged working professionals (healthy volunteers taking part in co-creative session with generative AI)	Low-medium: peer-review process unclear, methods and study design not described adequately enough to replicate (e.g., recruitment of participants, data collection)	- AT in Korean context: “the act of applying ink contributes to finding emotional stability and introspection” - Focus on “appreciating artworks” rather than artmaking	- “Generative AI-based traditional Korean painting experience” [text-to-image] - A GAI (generative AI) tool “trained in Korean painting” - “Co-creation process with GAI, introducing elements of randomnes, surprise, and mimesis into artworks”	- To “achieve psychological wellbeing during the creation or appreciation of [AI-generated] artworks,” catharsis and user satisfaction - Features of text-to-image GAI indicated as potentially therapeutic: (1) intentional imperfection (“may be perceived as more beautiful and authentic than perfection, fostering a sense of observation through which individuals can find healing”), (2) serendipity [“(AI-generated) results often surpass our imagination or expectations, producing unintended artworks. This can evoke a wow effect and enhance the emotional state of the viewers”]
(8) Pease et al. (2022) UK / USA / Ireland	Conference proceedings: 13th International Conference on Computational Creativity (ICCC 2022), Bolzano, Italy	To discuss “therapeutic modalities through the lens of computational creativity and explore opportunities in this exciting emerging domain”	M: literature review of “creativity software” applications in AT, vignettes, set of recommendations for therapeutic computational creativity (TCC) P: discussion between four authors: two CC researchers and two psychotherapists	Medium-high: peer-reviewed publication, good understanding of CC for therapeutic applications	- Focus primarily on AT, with references to other arts therapies, psychotherapy and occupational therapy - Clear distinction between AT and “everyday therapeutic art” - References to selected AT approaches, like Kramer’s “third hand” (analogy to AI’s potential role)	- Therapeutic computational creativity (TCC) as “an emerging sub-domain of CC that studies creative systems that promote wellbeing”	- Co-creative systems can “offset or even eliminate the need for any artistic expertise (…) and as such extend creative self-expression” - Wide potential application of TCC: “from casual wellness applications to improve mood, to the potential to be incorporated into treatment of conditions such as depression, anxiety, bereavement and trauma,” and wide range of “artistic modalities” - Analogy between TCC systems and the “third hard” concept (i.e., supporting client’s self-expression by gently assisting/guiding the creative process)
(9) Yilma et al. (2024) Luxembourg / Netherlands / Canada	Conference proceedings: CHI Conference on Human Factors in Computing Systems (CHI’24), Honolulu, USA	To investigate the potential of Machine Learning (ML) based Visual Art Recommendation System (VA RecSys) “to enable personalized therapeutic visual art experiences for post-ICU patients”	IP: patients with post-intensive care syndrome (PICS) M1: development study with usability testing, interviews P1: 4 experts: ICU nurses, healthcare and affective design researchers M2: pre-post study (guided AT session): reflexive thematic & sentiment analyses, psychometric testing P2: 150 post-COVID patients from UK, USA and South Africa	Medium-high: peer-review process unclear, methods clearly described, sound analysis and implications for practice	- AT “as an umbrella term where art serves as a medium for therapeutic benefits,” including “engaging with existing artwork to stimulate emotions and self-reflections” and “use of visual art as a positive distraction” (i.e., [art] exposure therapy)	- Machine Learning-based Visual Art Reccommendation System (ML-based VA RecSys): “personalisation of healing art for therapeutic purposes” - Proposing “personalized guided art therapy”: choosing preferred artworks from sample paintings, followed by AI recommending artworks and “asking questions that encourage participants to engage with the paintings,” and finally reflecting on the experience	- To “improve temporary stress and evoke positive emotions” for post-ICU patients - “A careful selection of paintings tailored to the individual patient speaks to their unique circumstances, fostering self-reflection and healing,” AI “can bridge the gap between the vast universe of artworks and the unique emotional needs of each patient” - Themes (“healing elements”) identified: hope and purpose, rejuvenation, engagement, safety, sensory pleasure, relevance, personal preference
(10) Yoo et al. (2023) USA	Conference proceedings: 11th International Conference on Affective Computing and Intelligent Interaction Workshops and Demos (ACIIW 2023), Cambridge, USA	To investigate “the potential of generative AI as an effective agent for conducting art therapy”	M: description of an app “which incorporates art therapy methodologies and generative AI technology” (methods not clear)	Low-medium: peer-review process unclear, sound description of the app with clear aims and functionality but no theoretical base or rationale provided	- Focus on “utilization of ‘Art’ as a therapeutic approach” and “the potential of generative AI as an effective agent for conducting art therapy” - Understanding of AT process unclear, collaboration with a “certified art therapist” mentioned but not details	- Mind Palette app utilizing GPT3 and Dall-E technologies (including “voice-based generative AI conversational interactions” and “AI-generated artwork recommendations”)	- To “facilitate discussions about emotions, encourage self-expression through art creation, and provide congnitive-behavioral therapeutic advice in both verbal and visual ways” - Recommendations for “specific artworks, colors, and images,” tailored to [users’] emotional state - “By actively engaging users in the drawing process and encouraging self-reflection, the AI agent creates a space for users to express their emotions artistically while gaining a deeper understanding of their own experiences”

### 2.4 Data analysis and synthesis

**Figure 2:**
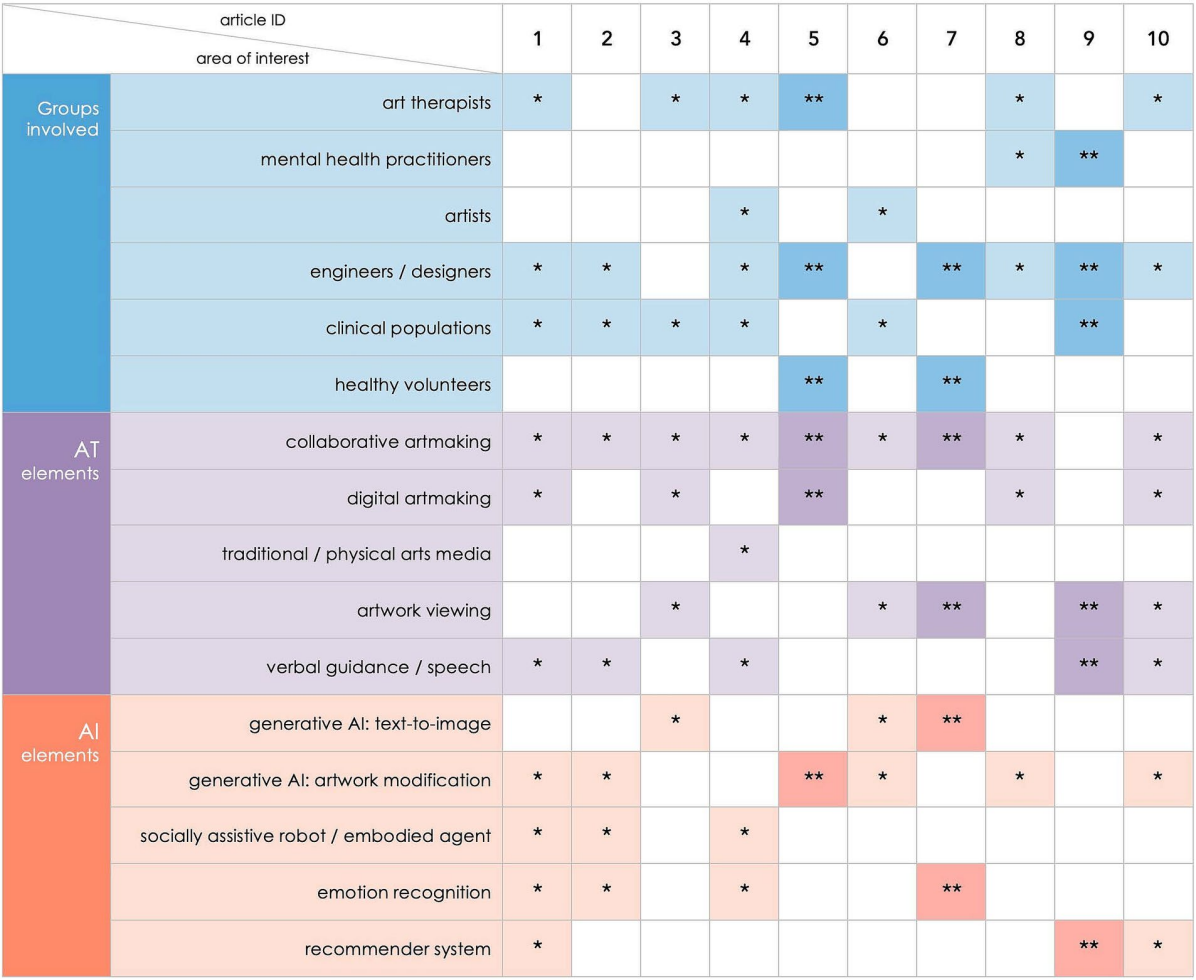
Selected characteristics of included papers, including groups of participants or stakeholders involved, elements of proposed systems or interventions relevant to art therapy, and types of AI technology referred to. *Indicates that a characteristic is present in a theoretical/opinion paper (i.e., in developmental stages of a prototype development or discussed within a literature review). **Indicates that a characteristic is present in an empirical study (i.e., has been applied in practice and discussed/evaluated).

The initial test searches revealed that some literature claiming to focus on art therapy lacked insight into the nature of art therapy practice. The opposite was at times true, with some texts making less direct links to art therapy transpiring to be indeed particularly relevant and grounded strongly in psychotherapeutic principles. This led us to consider a “layered” review, with texts grouped according to their relevance to the discussion on the links between AI and AT. However, on closer examination of the literature, we realized that adopting categories of any sort would oversimplify the complex and hugely differing characteristics of papers emerging as relevant. Instead, we opted for a “matrix” approach to classifying identified literature, which allows for multiple groupings based on characteristics, such as stakeholders involved, art therapy elements discussed, or AI elements examined (see Figure 2). A matrix allows for non-exclusive and overlapping categories to emerge, which more accurately resembles the nature of the literature reviewed. A similar approach has been previously successfully adopted in our related review (Zubala et al., 2021).

**Figure 3:**
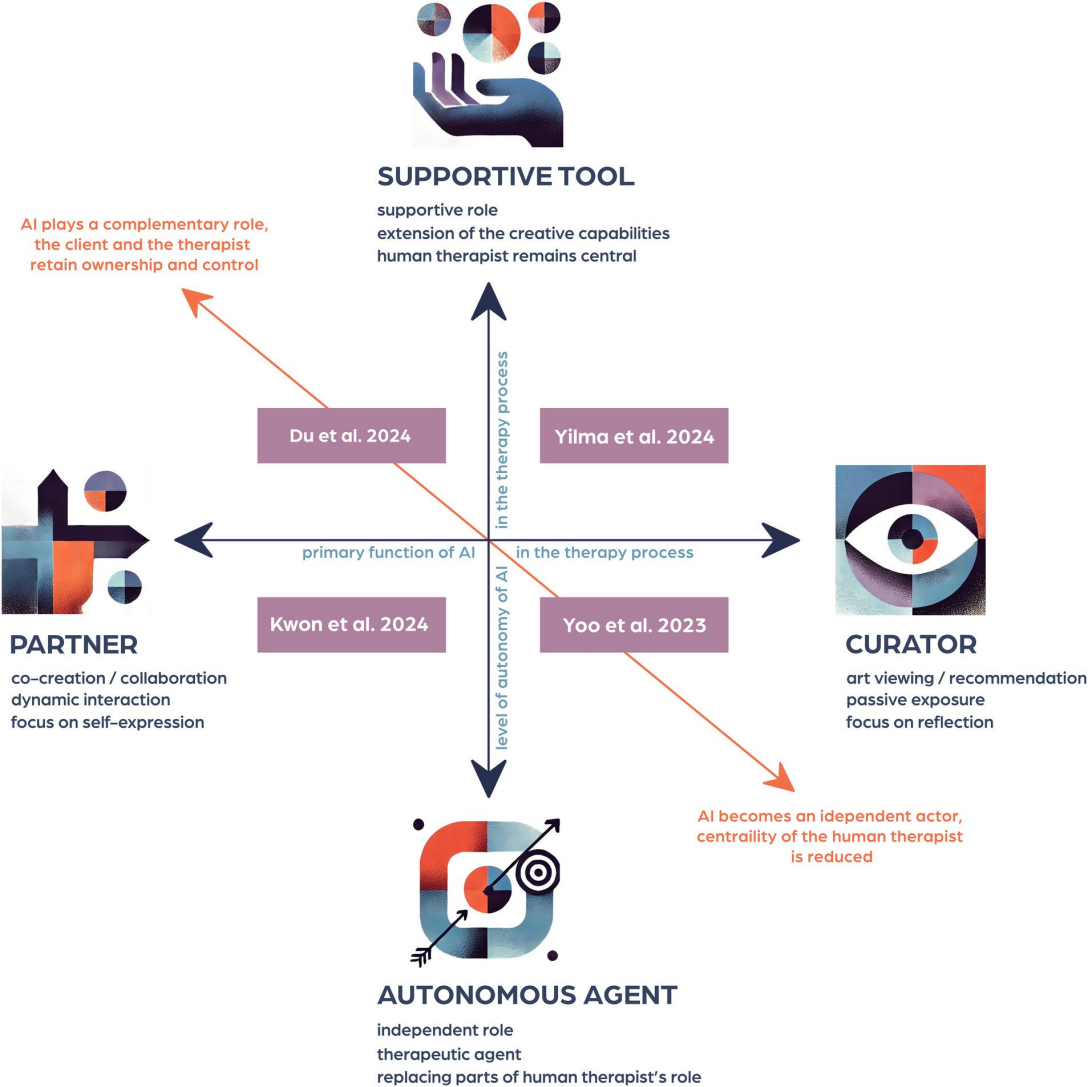
Positioning diagram of the role of AI in AT process, with examples of papers corresponding to the four quadrants (Visual icons co-created with ChatGPT-4o, OpenAI).

Following initial data extraction, the characteristics of included papers were tabulated (see Table 1) and subsequently repeatedly examined in depth to reveal recurrent themes emerging across the literature. Particular attention was given to identifying how AT and AI were contextualized within each paper and how the authors perceived the role of creative AI in AT practice (including potential wellbeing benefits and therapeutic features). Further relational analysis of the themes identified particularly strong associations between some of them, which we eventually captured in a diagram of two interrelated dimensions (discussed later), key for the application of AI in AT contexts (Figure 3). Discussion with the PPI group on the relevance of emerging themes for mental health community helped us refine them further and allow the opportunity to consider the perspectives of the people with lived experience of mental ill health in this exploratory stage work. An infographic capturing the key findings was also produced for wider dissemination purposes.

### 2.5 Notes on the cross-disciplinary search strategy and excluded papers

The inclusive transdisciplinary approach to literature searching intended to reflect the research practices in both disciplines (appreciating the challenging implications for analysis/synthesis). From our initial searches we soon realized that by following either of our disciplines’ usual approach, we would likely miss relevant literature and would not be able to provide a balanced perspective on the current state of knowledge. We settled for certain strategies from either of the disciplines which we believed would allow us to best meet the review aims. Therefore, although conference proceedings are traditionally excluded from reviews in health due to an often less-rigorous (or indeed absent) peer review process, they were considered for this work, as would be the standard practice in the field of computing. Our reasoning was that conference papers were likely to represent the most recent developments in the fast-changing interdisciplinary area we were focusing on, which the reviews within traditional psychotherapy and mental health paradigms might possibly miss or discover with a delay. The downside to our approach was an increased (in relation to a more standard health-oriented review) occurrence of papers of insufficient academic merit among the records identified, and of papers with lesser relevance and impact. Consequently, decisions on inclusion of individual papers were far from straightforward and the process took significantly longer than in case of a more standard review, including only peer-reviewed publications.

A single particularly prominent challenge in considering papers for inclusion was often drastically different conceptualisation of art therapy, including vast differences among the authors’ understanding of its nature, key principles and its place within the wider context of mental health treatment. While we anticipated that the papers might adopt quite diverse definitions of art therapy, not necessarily exact to those of AATA and BAAT, it was unquestionable to us that the key humanistic and therapeutic principles of art therapy need to be reflected in the authors’ portrayal of the discipline, in particular the centrality of the therapist’s role in supporting clients in their process of therapeutic change. Several papers were excluded due to a particularly flawed perception of art therapy as a practice of interpreting clients’ artwork in an authoritative way or assessing the clients’ artistic outputs. On rare occasions, papers were excluded due to positioning themselves as seemingly relevant to AT practice and in fact failing to demonstrate any evidence of being rooted in AT principles. It is important to note that such papers have been published and should therefore be particularly closely scrutinized by researchers and practitioners encountering them in terms of their value (or indeed detriment) to the building of knowledge in an emerging field.

## 3 Findings

### 3.1 Characteristics of included papers

The final searches took place in July 2024. Ten papers were included in this review (see Table 1), of which four were peer-reviewed journal articles and six were excerpts from conference proceedings (with peer-review process unclear in most cases). While most papers included mixed methodologies, three reported on empirical studies, four were primarily literature reviews, and three were accounts of early prototype development (with no actual product developed and no testing element included). Confidence in the studies’ findings (in case of experimental research) or authors’ opinions (in case of theoretical papers) was established at between low-medium for three papers to high for two other papers, with two papers considered of medium and three papers of medium-high quality.

The authors of included papers were based in different countries across several continents, as were the conferences at which some of this work was presented. The earliest included paper was from 2018 and six were published only within the last 2 years prior to the current review, indicating the rapidly growing interest (and knowledge) of the subject in the research community. As predicted, there were no relevant papers identified that would pre-date to more than several years in the past.

While all included papers focused on applicability of AI to therapy (and therefore indirectly pointing to its use with clinical populations), only three focused on specific clinical conditions, including people living with dementia (Harwood et al., 2019), people with complex communication needs (AlSadoun et al., 2020) and patients with post-intensive care syndrome (Yilma et al., 2024) (Figure 2). One review intended to focus on older people specifically (Bossema et al., 2023) but failed to identify studies directly relevant to this age group. Of the three empirical studies, only one included participants with a clinical condition (patients with post-intensive care syndrome, Yilma et al., 2024) and the remaining two included healthy adult volunteers. The majority of the papers (eight out of ten) included in this review recognized engineers, computing specialists, software developers and/or designers as a group essential to be included in conversations and the process of implementing the use of AI in therapy. Two papers (Cooney and Menezes, 2018; Harwood et al., 2019) mentioned collaborating with artists. Six papers recognized the need for including art therapists as stakeholders in prototyping and/or discussions, of which one demonstrated evidence of the actual collaboration with art therapists in practice (Du et al., 2024). Another empirical study included mental health practitioners instead (Yilma et al., 2024).

Heterogeneity of included papers was evident not only in the wide range of methodologies involved (often within individual papers), but also in the level of detail in reporting and the actual depth of investigations undertaken. While some of this work required undoubtedly complex background research and detailed analysis, resulting in a significant contribution to knowledge beyond a singular study (e.g., Cooney and Menezes, 2018; Du et al., 2024), other papers focused very specifically on a single point in time or a specific product (e.g., AlSadoun et al., 2020; Yoo et al., 2023), limiting applicability of the research to wider contexts. Papers also differed in depth of analysis and understanding of both the art therapy principles and technological potential, resulting in more or less clear implications for practice. Stages of investigations also differed largely, from conceptual developments (e.g., Harwood et al., 2019), through early prototyping (e.g., Yoo et al., 2023) to a more structured testing of early interventions (Du et al., 2024). Importantly, none of this research reached a stage of effectiveness assessment or implementation in practice.

#### 3.1.1 The nature of art therapy approaches in included papers

The included papers differed in how they perceived art therapy or indeed which aspects of art therapy they considered particularly suited for the application of creative AI. Two papers referred to art therapy within the Expressive Therapies Continuum model (ETC) specifically (Choe and Hinz, 2024; Du et al., 2024), while some papers adopted a much broader stance, distancing themselves (intentionally or not) from more defined therapeutic approaches, and at times in fact sketchily positioning art therapy either as an “an umbrella term where art serves as a medium for therapeutic benefits” (Yilma et al., 2024, also similar in Harwood et al., 2019; Yoo et al., 2023) or as part of a wider field concerned with therapeutic applications, such as (psycho)therapy (Pease et al., 2022) or arts and creativity (Bossema et al., 2023). Some authors have demonstrated their understanding of art therapy more clearly than others, particularly in relation to definitions of American Art Therapy Association (2024) and British Association of Art Therapists (2024a, 2024b), referring more directly to the central role of the therapeutic relationship and process (Choe and Hinz, 2024; Du et al., 2024; Pease et al., 2022). In some instances, the authors’ views on core to art therapy mechanisms were less clear or simply not discussed with enough detail (e.g., AlSadoun et al., 2020; Yoo et al., 2023). Nevertheless, despite those differences in understanding and focus, on close examination all included studies shared an underlying view of art therapy as a psychotherapeutic modality, aiming to increase wellbeing and/or treat mental ill-health (which was the basis for their inclusion in the current review, as discussed previously). Two papers focused on very specific geographical and cultural contexts (Saudi Arabia – AlSadoun et al., 2020, South Korea – Kwon et al., 2024), indicating expected differences in their understanding of art therapy in relation to traditionally Western definitions (for example, including more structure and culture-specific artmaking techniques – to be discussed later in more detail). Although rarely explicitly specified, all papers seemed to focus on individual (one-to-one) therapy, either involving human art therapists or using an AI system in place of a human therapist.

All included papers except one (Yilma et al., 2024) focused, at least to some extent, on collaborative artmaking in the context of AI-mediated art therapy, i.e., co-creating artwork with the participation of both human and AI (see Figure 2). Yilma et al. (2024) focused instead on AI-generated artwork being viewed by human participants. In fact, art viewing played a significant role in half of the papers in addition to co-creation. Digital artmaking (by human participants) featured explicitly in half of the included papers, with the remainder proposing a more passive role of human participants as viewers or prompt generators, with a noticeable distinction of Cooney and Menezes (2018), the only paper focusing specifically on painting/drawing using traditional physical arts media. Five papers featured speech or text-based elements in addition to artmaking and/or AI-assisted image generation – either in the form of guidance from the AI system or (optional) verbal human-AI interaction.

#### 3.1.2 The nature of creative AI technologies in included papers

Some papers referred to multiple forms of AI-based technologies, either blending them within one intervention (Kwon et al., 2024; Yoo et al., 2023) or theoretically exploring a variety of AI-based applications in art therapy context (AlSadoun et al., 2020; Bossema et al., 2023); others focussed on more specific systems or functions (e.g., Yilma et al., 2024; Choe and Hinz, 2024; Du et al., 2024; Pease et al., 2022). Image modification with the use of generative AI featured most frequently in the papers, often implying human-AI co-creation and multiple instances of modifications applied by both partners in the artmaking process. Some papers referred directly to text-to-image generative AI technologies (particularly Choe and Hinz, 2024), picturing a unique type of co-creation in which a human participant communicates primarily via verbal/text prompts and creative AI system responds with visuals. Several papers referred to the possibility of introducing socially assistive robots into therapy spaces, with Cooney and Menezes (2018) imagining a bespoke AI-based embodied agent for use in art therapy specifically. Emotion recognition is a notable feature of some of the discussed AI-based systems, particularly in Kwon et al. (2024) and Cooney and Menezes (2018). Recommender systems are another important technology, featuring most strongly in Yilma et al. (2024) and Yoo et al. (2023).

### 3.2 Key themes of significance for AT practice

Alongside more general issues around (digital) data protection and safety of personal information featuring to varying extent in the majority of included papers, the literature lists a number of AI features that might indeed present relevance to the therapy process, but are not unique to AI-mediated therapy, including opportunities for personalisation and responsiveness (e.g., Bossema et al., 2023; AlSadoun et al., 2020). For example, real-time feedback is traditionally provided by human art therapists in therapy not involving AI, personalisation or tailoring of treatment is a common practice and a part of the role of an art therapist.

The following section focuses on several recurrent themes identified across the papers, with direct implications for art therapy practice, including such features of AI that provide genuinely novel qualities in this context. They are intended to be seen as areas for consideration when designing AI-mediated AT interventions. Some themes are recommendations for practice in its nature while others indicate significance of a certain concept but also recognize the potential ambivalence of its impact on practice and/or the therapy process. Whenever relevant, themes from the literature are supplemented by corresponding insights from the PPI group.

#### 3.2.1 Including art therapists in the design process

Several papers recognize that involving art therapists should be integral to the process of conceptualizing, designing and prototyping AI-based technologies for use in therapy, ensuring their therapeutic relevance. A similar suggestion was made by a member of the PPI group, who also felt that professionals of related disciplines (e.g., occupational therapists) might be able to provide valuable insights. Pease et al. (2022) discuss the necessity of engaging psychotherapists in designing therapeutic computational creativity systems and argue that art therapists’ insights are vital to ensure that AI systems can support clients’ creative and emotional needs. Choe and Hinz (2024) highlight the responsibility of art therapists to critically assess and contribute to how AI is integrated into art therapy, ensuring that AI aligns with its values and is used in ways that enhance, rather than replace, human creativity. The author’s stress that art therapists should guide ethical considerations and the therapeutic uses of AI in creative processes (Choe and Hinz, 2024).

Of the projects that reached prototyping stages, only one provided evidence of collaborating with art therapists across the design phases, involving five experienced registered art therapists throughout the iterative design process lasting over 10 months (Du et al., 2024). Two empirical studies did not mention involving art therapists at all (Kwon et al., 2024; Yilma et al., 2024), while Yoo et al. (2023) reported collaborating with a certified art therapist with no further details provided. AlSadoun et al. (2020) propose to include art therapists late in the design process (at a point of prototype evaluation), but specifically highlight the importance of (local) art therapists in ensuring that AI-based systems are culturally relevant.

#### 3.2.2 Cultural adaptation and sensitivities

Some authors emphasize the importance of considering cultural sensitivities and adapting AI-supported interventions to cultural contexts, stressing that AI-mediated art therapy must reflect and respect clients’ cultural backgrounds, artistic traditions, and personal values. Choe and Hinz (2024) in particular warn that using “imbalanced or flawed datasets” to train AI models could result in amplified systemic biases, cultural nuances being lost and specific artistic styles favored, with potential detrimental effect on the art therapy process. An art therapist participant helping to inform the AI-mediated intervention developed by Du et al. (2024) pointed to the cultural significance of art materials and artistic styles for AT practice. This prompted the authors to highlight the value of training the AI-based models in diverse painting styles originating in different countries and not in the Western context exclusively (Du et al., 2024).

AlSadoun et al. (2020) explicitly focus on introducing AI to art therapy in the context of the Arab region, emphasizing the need for culturally sensitive approaches and recommending involving local therapists, designers, and developers in the process. Kwon et al. (2024) highlight the importance of cultural identity and heritage and explore how AI can be used to preserve and integrate traditional cultural elements in AT context. The authors propose that incorporating traditional art forms, such as Korean painting, into AI-mediated art therapy can foster a deeper connection with cultural roots, enhancing the psychological benefits of therapy, and increasing client satisfaction with the process (Kwon et al., 2024). In addition to the geographical contexts, Harwood et al. (2019) focus on the need for AI-mediated art therapy to account for the social and cultural needs of aging populations and point to the unique visual characteristics of some of the AI-generated art (referred to as “psychedelic and fantasmorganic” properties) as particularly interesting and potentially therapeutic for people with dementia.

#### 3.2.3 Inclusivity and accessibility

Some papers discuss the notion that the use of AI can help make art therapy more inclusive. By lowering the expertise threshold, AI tools could enable participation in artmaking for individuals across a spectrum of (real or perceived) abilities and skills, including people with physical, cognitive, or communication challenges (Choe and Hinz, 2024; Du et al., 2024). AlSadoun et al. (2020) propose that AI-based tools can accommodate users with communication or developmental disabilities, allowing them to engage in therapy in ways that are tailored to their specific needs. Pease et al. (2022) discuss how co-creative AI systems can lower barriers to artmaking by supporting individuals who do not see themselves as creative, fostering a sense of achievement and enjoyment of the creative process.

In addition to the potential for “democratizing artmaking” (Choe and Hinz, 2024), AI-based technology has also been proposed to expand access to therapy, enabling scalable interventions outside of traditional therapy spaces, and potentially addressing gaps in mental health provision, particularly in underserved or remote communities (Yoo et al., 2023; Yilma et al., 2024; Pease et al., 2022; Cooney and Menezes, 2018).

#### 3.2.4 Impact on creativity and self-expression

The reviewed literature generally agrees that AI can assist users in the creative process and “enrich the expressive repertoire” (Du et al., 2024). AI-based tools allow for experimentation with new forms of self-expression and exploration of creative possibilities that might otherwise be inaccessible, easing the constraints of perfectionism, fear of judgment, or the initial hesitation about engaging in artmaking (Du et al., 2024; Pease et al., 2022).

At the same time, several papers express concern that creative AI might make the artmaking process “too easy” or the resulting artwork too “sophisticated,” which might be perceived as overtaking the creative process (Pease et al., 2022; Du et al., 2024; Choe and Hinz, 2024). Du et al. (2024) acknowledge that the power of AI to generate sophisticated images in a short time and with little effort on the human co-creator’s side can feel overpowering and may be in fact detrimental to self-expression. Some authors refer to the idea of struggling through the creative process as essential for personal growth in art therapy and wonder if its therapeutic value might be diminished if AI is integrated into the process (Du et al., 2024; Pease et al., 2022; Choe and Hinz, 2024). Pease et al. (2022) cite Csikszentmihalyi’s idea of “being stuck” as a desirable part of the creative flow and suggest that AI-based technology for art therapy should account for this important dynamic.

Recognizing the potentially ambivalent impact of AI-based technology on self-expression, Du et al. (2024) conclude that it is possible for AI-based technologies to “ease and augment the creative expression (…) without taking over the whole art-making process” (Du et al., 2024). Choe and Hinz (2024) also acknowledge AI’s “capabilities beyond those that conventional art media can provide,” while indicating the risk that “over-reliance on AI tools may (…) reduce expressive authenticity and limit artistic intuition.” Our PPI group have raised similar concerns, discussing at large that AI-supported AT interventions can and should enhance creative and therapeutic processes, and not attempt to replace them.

#### 3.2.5 Unpredictability and imperfection

Several papers refer to unpredictability, randomness, imperfection, and serendipity as qualities associated with AI-generated (or co-created) art, and these characteristics are suggested to be influencing the therapy process in various ways.

Kwon et al. (2024) propose that imperfection and serendipity introduced by generative AI in artwork can be positively surprising and enhance user satisfaction during the co-creation process, extending to therapeutic benefits such as achieving psychological catharsis. Yoo et al. (2023) seem to refer to similar ideas in stating that generative AI can “provide ambiguous situations and conversations,” which they suggest resembles a process of human-human therapeutic interaction.

Du et al. (2024) notice that unpredictability in AI-enhanced artmaking “creates space for exploration and creativity,” can be enjoyable, and allow for creative engagement with the common in psychotherapy theme of tension between control and lack of control. Two of the art therapist participants in this study referred to this quality of AI as potentially helpful for clients who would benefit from working with the sense of surrender and toward letting go of control. The authors also seem to suggest that the unpredictability of AI tools could allow the therapy process to progress faster due to bypassing the common tendency of clients to be more controlling at the start of their therapy, when using physical art materials (Du et al., 2024). Recognizing the potential therapeutic benefits of “playing with unpredictability,” Du et al. (2024) also highlight that this is not always desirable in therapy context, which designers should be aware of.

### 3.3 Positioning diagram of creative AI in AT process

The papers describe the potential role of AI in the AT process in a variety of ways, emphasizing how AI can augment, enhance, or transform the therapeutic experience. We propose that the position of creative AI in the therapy process can be mapped out on a diagram of two dimensions, one being a spectrum of the type of creative involvement from AI, between co-creation and art viewing or recommendation, and the other a spectrum of AI’s level of autonomy in the therapy process, ranging from a supportive to a fully independent role (Figure 3). According to this, AI can take a variety of roles in art therapy, being a partner or a curator in the creative process, and being a supportive tool or an autonomous agent in the therapy process.

AI’s roles are conceptualized here on dimensions rather than as categories, since they are not necessarily fixed within a single intervention or even a single session, and it is possible for AI-based systems to move fluidly between them. For instance, AI might act as a supportive tool when co-creating art and shift to a more autonomous role when offering feedback or analysing emotional cues.

An additional diagonally placed dimension represents two extremes: AI in a complementary role, as a purely supportive tool assisting in co-creation, with the client and therapist retaining full ownership and control of the creative and therapy processes on one end of the spectrum, and AI as an independent actor, capable of making decisions about art recommendation, emotional guidance, and even therapeutic interventions without human input on the opposite end of the spectrum.

The diagram enables mapping of any AI-enhanced arts-based therapeutic intervention across the four quadrants representing varying levels of AI’s autonomy and involvement in the creative process. The following section describes the proposed dimensions in more detail and section 3.4 provides examples from the papers that focus on interventions representative of the four emergent quadrants.

#### 3.3.1 AI as partner vs. curator

Most of the papers in this review focus on co-creation (i.e., human-AI collaboration) while several papers focus primarily on viewing/appreciating artwork generated or recommended by AI and some consider both human-AI co-creation and art viewing in the same intervention (see Figure 3). We note that art viewing, while potentially therapeutic, is not typically consistent with art therapy practice. However, for the purpose of this exploratory review, it is included here as a potential element within art therapy, provided that it is incorporated in the therapy process with a clear therapeutic intention, e.g., to inspire self-reflection or evoke a feeling leading potentially to a therapeutic insight.

##### 3.3.1.1 AI as partner (AI-human collaboration)

In this scenario, AI acts as a co-creator, a partner in the collaborative creative process. It assists therapy clients in (typically digital) artmaking, providing AI-based tools, generating visual images in response to verbalized ideas, or modifying the artwork in real time. The client is similarly active in the co-creation process and in verbal/text-based or visual exchange with AI. This dynamic interaction promotes self-expression, by supporting clients to explore their creativity and new artistic possibilities.

AI is framed as a collaborator that helps extend the user’s creative abilities in a paper by Pease et al. (2022), and Cooney and Menezes (2018) similarly refer to their proposed art therapy robot as an “interaction partner.” Choe and Hinz (2024) generally seem to position AI as augmenting human creativity, but also acknowledge that its generative capacities bring it closer to a co-creator in AI-human creative interaction.

##### 3.3.1.2 AI as curator (AI-to-human recommendation)

In this scenario, AI selects (or generates) and recommends personalized artwork based on users’ emotions, current sense of wellbeing or preferences. Through generating artwork for clients to view and engage with, the AI functions as a curator that tailors the therapeutic experience. The client does not actively participate in the creation process but is encouraged to reflect on AI-generated imagery, working toward personal therapy goals. As a recommender, AI could facilitate and support reflection by interacting with clients through text-based or verbal conversations and/or via visual cues.

Prototypes for AI-based recommender systems for use in AT proposed by Yoo et al. (2023) and Yilma et al. (2024) present users with personalized artwork and facilitate discussion, promoting engagement with the selected image and active reflection.

#### 3.3.2 AI as autonomous agent vs. supportive tool

Some papers propose that creative AI should be seen as a purely supportive tool enhancing human-led therapy and the client’s ability to express themselves creatively, while others suggest that AI may be closer to being an autonomous agent, capable of taking a leading role in complex interactions. In the first instance, human art therapists remain central in guiding the therapy process and ensuring that the AI system is used purposefully to meet therapeutic goals, while in the latter case, creative AI is seen as capable of replacing at least parts of the human therapist’s role.

Choe and Hinz (2024) argue against “oversimplifying AI generative art programs as just another tool” in the light of creative AI’s unique impact on aspects of key importance to AT, such as authenticity and ownership over the creative process and the artwork. Two other papers point briefly to the importance of ownership (Du et al., 2024; Cooney and Menezes, 2018), which was also mentioned by our PPI group members who felt that retaining ownership and a sense of agency was essential for both clients and therapists engaging in AI-mediated AT.

##### 3.3.2.1 AI as supportive tool

In this scenario, AI plays a supportive role, extending the user’s creative capabilities without taking over the creative process. The human therapist remains central, and AI is integrated as a tool to facilitate therapy.

Recognizing that AI can supplement and even enhance therapy, some authors still emphasise the presence of human art therapists as essential in guiding the therapy process, offering authentic empathy and therapeutic alliance (Pease et al., 2022; Choe and Hinz, 2024; Cooney and Menezes, 2018). Pease et al. (2022) refer to the Rogerian concept of bearing witness to the client’s process and holding space for the client as important therapeutically skills which cannot be realistically achieved by AI. Cooney and Menezes (2018) stress that their proposed art therapy robot should not replace human art therapists (except when no human therapists are available), which “might limit the depth of emotional engagement and the ability to navigate complex emotional landscapes,” and is instead envisioned as supporting therapists in their role. It is, however, not just a tool either, being actively engaged in shaping the therapeutic experience, guiding the clients through emotional and creative processes, and may therefore be simultaneously seen as a more independent agent (Cooney and Menezes, 2018).

##### 3.3.2.2 AI as autonomous agent

In this scenario, AI is envisioned to operate independently of human art therapists during the actual therapy sessions, being able to guide the client through creative process and reflection. This represents a major shift in the therapeutic dynamic, reducing the centrality of the human therapist.

AlSadoun et al. (2020) propose to only include art therapists at the intervention design stage and imagine an AI-driven embodied agent to subsequently act as an “assistant art therapist,” with no further input from human therapists necessary. Some authors suggest that autonomous creative AI would be able to interact without fatigue, judgment or bias, potentially creating a therapeutic space in which clients may feel safer to express themselves freely, particularly if they feel uncomfortable or vulnerable in human-human therapeutic relationship (Cooney and Menezes, 2018; Yoo et al., 2023).

### 3.4 Example papers corresponding to the four quadrants on the positioning diagram

#### 3.4.1 AI as partner and supportive tool

Du et al. (2024) introduce an AI-infused artmaking system assisting clients in creating artwork collaboratively, enhancing their sense of agency and promoting creative self-expression. AI supports the clients by lowering barriers to creative expression, augmenting their capabilities, or introducing novel elements, without making autonomous decisions. While AI helps in facilitating the artmaking process and promoting the emergence of insights through the co-creation process, the user maintains creative control.

#### 3.4.2 AI as curator and supportive tool

Yilma et al. (2024) demonstrate an AI-based system personalizing art recommendations for post-ICU patients based on their emotional state and psychological profile. Viewing AI-recommended artwork serves as a form of exposure therapy, where users engage with emotionally resonant images to improve their wellbeing. AI functions as a curator of personalized art selection, providing supportive visual prompts for the user to reflect upon, but does not actively engage in generating or interpreting the creative content.

#### 3.4.3 AI as curator and autonomous agent

Yoo et al. (2023) introduce a mobile application that combines AI-generated art recommendations and interactions with an AI chatbot, aiming to guide clients through self-expression and reflection on their emotions and providing them with cognitive-behavioral advice. Generative AI bases its guidance on its interpretation of user’s emotions and needs, and plays a decisive role in driving the therapeutic process, requiring minimal input from either the user or the therapist.

#### 3.4.4 AI as partner and autonomous agent

Kwon et al. (2024) examine the application of AI in generating traditional Korean paintings for therapeutic purposes. The focus is on the co-creation between AI and the user, with no specific mention of the need for human therapist involvement, implying that AI can deliver the therapeutic experience independently. While AI and the user collaborate creatively, AI plays a more active role, generating the artwork and introducing unexpected or serendipitous elements into the process. AI is positioned as a therapeutic agent facilitating emotional stability and psychological improvement through the co-creation of art.

## 4 Discussion

The premise for this review was based on a notion that a truly collaborative dialogue between the fields of computing and psychotherapy is needed for novel, safe and person-centered AI-assisted solutions for mental ill-health prevention and treatment. For art psychotherapy practice this demands assessment and understanding of the opportunities and risks presented by creative AI in particular. To our best knowledge, this is the first publication to explicitly bring the two areas together and as such has an exploratory character, proposing foundations for future more focused work.

This integrative review was designed to be responsive to our knowledge growing with its development and new research emerging. While the research questions posed at the beginning remain valid, new priorities for investigation and synthesis were revealed in the configurative process of our data analysis (Gough et al., 2012). This led us to propose a framework for positioning creative AI technologies in relation to the creative and therapeutic processes in art therapy, referred to earlier as a positioning diagram (Figure 3) - hopefully a useful tool for making sense of the diverse solutions and ideas we can expect to rapidly develop and demand critical evaluation of their potential for applicability. This review has also identified several themes of key importance to practice, covering concepts such as (co)creativity, inclusivity, ownership, control, therapeutic relationship and ethics. We will now provide more context for some of these, not as an exhaustive analysis but rather to invite discussion beyond this paper.

### 4.1 Relevance and dynamic growth of research

The wide geographical spread of the early literature exploring the connections between creative AI and art therapy reflects the growing interest in the application of AI in therapeutic contexts across the globe. The majority of the research efforts so far are in conceptual or early developmental stages at most and we do not yet have trustworthy evidence or demonstrated benefits of AI-mediated art therapy, except for rare anecdotal case studies. For example, beneficial effects on mood and engagement were reported from an AI-assisted creative process of reminiscing for clients with dementia (Lee, 2023). Progress in research can be, however, expected immediately, and two very relevant studies have been published recently that contribute some understanding of art therapists’ perspective on AI, based on interviews with nine American art therapists (Shojaei et al., 2024) and a survey of 56 art therapists registered in the USA, Canada, Australia and UK (Jütte et al., 2024). The latter study is a good early example of research moving beyond building general foundations and into more specific applications – in that case, interested in utilizing AI-generated art from medical images in art therapy for melanoma patients. While recent research has also provided some insight into clients’ attitudes and expectation of AI-mediated psychotherapy (Aktan et al., 2022), none has yet emerged in art therapy specifically.

The art therapy community needs to be mindful that among genuine research efforts, as in the examples above, there are instances of research studies which are misleadingly positioned within an area of inquiry and practice that they do not belong to, such as a recently published systematic review in which none of the included papers focus on art therapy despite a deceitful title, promising to discuss the “emergence of artificial intelligence art therapies” (Luo et al., 2024). Such publications should be distinguished from those that might unintentionally misinterpret AI’s role in AT due to misunderstanding of the nature of AT practice (e.g., Cheatley et al., 2022 inferring the unique value of computational creativity systems in enabling self-expression for clients with no artistic training or expertise, based on an incorrect assumption that artistic skills are required to be able to engage in art therapy). Due to existing preconceptions or inaccurate perception of art therapy, the relevance of any proposed AI-enhanced solutions should be considered with caution and against the foundational principles of this unique profession.

### 4.2 New modes of practice

The recent spectacular growth of generative AI unlocks new and innovative ways of practicing art therapy, potentially highly personalized and perhaps more accessible in certain circumstances. Possibilities include incorporating co-creative software into synchronous and asynchronous therapy situations, also in group therapy and remote therapy, as well as reaching new populations and extending provision of art therapy to those unable to access services or use traditional arts media.

It is being proposed that implementing AI in psychotherapy interventions might benefit remote and rural populations with limited access to therapy (Miner et al., 2019) or clients who may experience traditional therapy as stigmatizing or embarrassing, or simply preferring “low-threshold interventions” (Fiske et al., 2019). Art therapy research so far has been particularly interested in therapeutic impacts of creative AI for older adults (Bossema et al., 2023; Harwood et al., 2019) and clients with disabilities and complex communication needs (Choe and Hinz, 2024; AlSadoun et al., 2020). While there might be some intuitive and conceptual indications for specific therapy settings or client groups to benefit from the use of AI, and suggestions of AI-enabled scalability of psychotherapy helping to fill gaps in mental health provision for underserved communities, research has not yet produced compelling evidence, and poses more questions than answers.

### 4.3 Implications for creativity and ownership

AI’s ability to generate images of artistic merit and to assist humans in the creative process challenges traditional concepts of creativity and ownership of artwork (Runco, 2023b). Creative AI certainly brings potential for therapeutic experiences not previously encountered in art therapy practice. AI’s ability to generate art autonomously or to dynamically co-create artwork with users introduces a new dimension to the creative process, enabling clients to experience a vast range of artistic expressions, and introducing an unprecedented level of creative freedom and playful exploration into the artmaking process (Garcia, 2024). AI-enhanced tools, filters and visual styles can transform artwork dramatically, introducing unexpected creative elements and directions, often beyond the user’s or therapist’s intention (Kwon et al., 2024; Du et al., 2024). The unpredictability and “controlled randomness” of AI-generated outputs provides a layer of novel (co-)creativity that users may find stimulating or reflective of unconscious processes. Imperfection of AI-generated art seems to mirror human creative flaws, potentially making the experience feel more authentic and personal.

However, AI’s ability to generate high-quality art in a short amount of time might undermine the therapeutic value of the “creative tension” (Zubala, 2018) and potentially diminish the sense of accomplishment that comes from engaging deeply with one’s own creative challenges. The sophistication of AI-generated art or perception that AI dominates the creative process can lead to client feeling disconnect and a reduced sense of ownership over their (co)created artwork. Achieving balance between easing the technical aspects of artmaking with AI while preserving user control and a sense of agency (for both clients and therapists) seems essential in art therapy context (Du et al., 2024; Cooney and Menezes, 2018), and can be ensured by only introducing AI-based elements that augment as opposed to replace human creative efforts.

### 4.4 New dimensions of therapeutic relationship

While they recognize that AI might be a useful addition to the therapists’ toolbox, psychotherapy scholars seem to share scepticism on its capability to act autonomously in the therapy context (de Mello and de Souza, 2019; Grodniewicz and Hohol, 2023; Fiske et al., 2019). Doubts have been expressed whether AI-mediated psychotherapy could ever be considered equal to traditional psychotherapy (Sedlakova and Trachsel, 2023), unless perhaps “psychotherapy in the future will mean something different than it means today” (Grodniewicz and Hohol, 2023). In theory, AI’s role as a “non-judgmental interactive agent” introduces a novel dynamic in therapy, with the potential for clients to feel more open to emotional expression and vulnerable disclosures when interacting with an AI agent that is perceived to lack the social biases and emotional limitations of a human therapist. Currently, the role of generative and conversational AI in psychotherapeutic processes seems to be, however, limited to supplementing human-delivered therapy (de Mello and de Souza, 2019; Grodniewicz and Hohol, 2023; Fiske et al., 2019; Haber et al., 2024), as it lacks the ability to form genuine relationships and to offer the kind of therapeutic presence that fosters personal growth. While AI may offer a degree of emotional responsiveness based on algorithms (Cooney, 2021), it cannot replace the human connection and authentic emotional engagement, navigate the complexities of ethical dilemmas or instigate the nuanced process of sense-making in situations involving trauma, grief, or emotional crises (Sedlakova and Trachsel, 2023). Since client expectations of therapy and trust in the therapy process largely influence clinical outcomes, preventing clients from forming inflated expectations of AI is ethically and clinically important, and its limited function in psychotherapeutic context should be transparently communicated to them (Sedlakova and Trachsel, 2023; Miner et al., 2019).

Therapeutic relationship (known also as therapeutic alliance) between the client and the therapist, which enables the psychotherapy process to unfold, is unlikely to be replicated in contact with AI (Fiske et al., 2019; Grodniewicz and Hohol, 2023) – at least in our current understanding of it, as an interpersonal phenomenon based on trust and empathy. However, in some contexts, there have been indications of AI users experiencing a sense of therapeutic alliance while interacting with chatbots, and questions posed if AI might in fact to be able to offer a valuable bond, potentially extending beyond the time the clinician is able to offer (Miner et al., 2019). Conversational AI on its own seems to be insufficient to inspire self-understanding and therapeutic change, but it might perhaps be able to adopt a “mediating role between a patient and a human therapist” (Sedlakova and Trachsel, 2023). Another supposition has been made that generative AI might inspire a more collaborative therapeutic relationship, empowering clients to engage more actively in their therapy (Haber et al., 2024).

Introduction of creative AI to art therapy space, with its unique qualities and potentially more tangible presence than previous technologies, inspires reflection on the multiple ways in which it can affect, or potentially even reconfigure, the therapeutic relationship. Conversational AI has been conceptualized by Haber et al. (2024) as “artificial third,” a new element in the traditional therapist-patient dyad, indicating the emergence of a distinctive triangular dynamic in (verbal) psychotherapy. The concept of a “third entity” within therapy space is, however, not new in psychodynamic tradition, and it takes a particularly tangible presence in the context of art therapy, in the form of an artwork complementing the triangular therapeutic relationship (Schaverien, 2000). Haywood and Grant (2022) have already projected that the therapeutic relationship might need to be redefined for digitally-mediated art therapy, proposing a model of hexagonal relationship, representing the new relational elements in the context of online art therapy. The inevitable impact of creative AI on art therapy practice seems to demand a dedicated discussion and perhaps a further rethink of the nature and shape of the therapeutic relationship.

## 5 Limitations

This review captures the state of the research on the application of AI to art therapy practice as of July 2024. Due to ongoing technological advancements and increasing interest in AI-based technologies for therapeutic purposes, we expect updates to the current review (or more focused knowledge syntheses to cover more specific aspects of practice) needed frequently for this work to remain current.

It is important to stress that ethical and privacy concerns over AI use in therapy, while hugely important, have been largely omitted in the current review. This is due to this exploratory work focusing primarily on themes and concerns specific to art therapy and creative AI. Responsible use of AI and its ethical and clinical implications demand dedicated investigation and a separate discussion (e.g. Flick and Worrall, 2022). It has already started in the wider area of psychotherapy, particularly concerning therapeutic relationship (e.g., Haber et al., 2024; Sedlakova and Trachsel, 2023), and should further develop in response to changes in technology and policies, as well as in the area of creative AI more specifically.

Perhaps the most obvious limitation, and at the same time strength, of this review lies in its inclusive approach toward publications that might typically be excluded from knowledge syntheses, such as conference proceedings and papers with unusually mixed methodologies (or indeed not subscribing to any particular methodology). We recognize that inclusion of non-peer reviewed papers might limit replicability of procedures. However, by representing the most current research directions and referring to novel ideas, such publications, in our view, offer value to reviews of exploratory nature, like this one.

## 6 Future directions

Given the preliminary nature of ideas and insights identified by this review and the potentially serious implications of introducing AI in art therapy, undoubtedly more exploratory research is needed, particularly involving art therapists, whose voice is currently largely missing in the literature. Unsurprisingly, notable and rare examples of research studies that genuinely include art therapists (e.g., Du et al., 2024; Shojaei et al., 2024) have led to insights into the role of AI in the therapy process which are richer and more relevant to practice. Understanding of the therapists’ perspective, reservations, and visions for the use of AI in practice is essential for any applications of real therapeutic value.

Currently, there is an apparent lack of ethical guidance and regulation on generative AI application to mental health treatment (Haber et al., 2024) as well as related training for health care professionals (Sedlakova and Trachsel, 2023). Future research must particularly thoroughly assess the risks of introducing AI in psychotherapy, including any potential clinical implications and areas of concern such as biases in generative models, limitations in privacy and confidentiality assurance, and potential dehumanization of the therapeutic relationship. Ethics-centered research should underpin evidence-based guidance on best practice in designing and implementing creative AI-based solutions with therapeutic intent. It is important that art therapists take active part in shaping those guidelines, certainly in the aspects uniquely relevant to arts-based therapeutic practice, such as authenticity of AI-generated art, safe storage of (digital) artwork, co-creation and ownership, the potential for creative expression with no physical presence or output, and ensuring that non-Western artistic traditions are not marginalized by AI models (Garcia, 2024).

In the meantime, we would like to encourage art therapy professional associations and training bodies to support therapists to cautiously and responsibly explore opportunities to adopt AI in practice, by offering suitable training and specialized supervision. Art therapists must proactively influence the introduction of creative AI within the clinical domain and have a challenging and unique role to play in managing its cultural and psychological implications (Choe and Hinz, 2024). Similarly, guidance is much needed for designers of AI-based systems on how to meaningfully benefit the therapeutic professions. Knowledge about different therapeutic traditions in the field of computing seems currently limited and is indeed essential for positioning AI in the therapy context, including in art therapy, or (potentially and cautiously) inspiring novel therapeutic modalities.

As for an immediate recommendation for research, we would like to highlight an urgent need to understand how the therapy process and the therapeutic relationship may be affected by introduction of creative AI into art therapy space. We also agree with previously recommended research directions into the impacts of AI on self-understanding, agency and identity (Fiske et al., 2019; Sedlakova and Trachsel, 2023). In the context of art therapy, such exploration will require a unique focus on the artwork and the creative process, adding complexity to the interactions and potential impacts of AI in the psychotherapy domain. Art therapy as a discipline is in a unique position to guide the expected therapeutic applications of creative AI, closely monitoring and evaluating its meaning for the therapeutic relationship.

Above all, further research should explore acceptability of AI-mediated therapeutic interventions for clients and patients. Understanding of the impact of AI applications on the therapy process, clients’ perception of it, their expectations of therapy and acceptance (or not) of its presence is crucial for any meaningful implementation, regardless of a seeming attractiveness or practical appeal of AI-based technologies. Transparency about what AI can and cannot achieve in a therapy situation is particularly important for clients, who should be supported in forming realistic expectations (Sedlakova and Trachsel, 2023). While creative AI, including the already impressive image-based generative AI, might present particularly attractive prospects, its real value for therapy can only be evidenced by and with clients, therapists, and the wider care team. Until then, its application to art therapy needs to be seen as speculative only.

## 7 Conclusion

This review offers insights into early research focusing on the application of AI-based technologies to art therapy practice, identifies key areas for consideration in development of AI-mediated interventions, and proposes a unique positioning perspective of the role of creative AI in the art therapy process.

The introduction of AI into art therapy space presents a range of exciting opportunities, from enhanced potential for creative expression, through to prospects of more inclusive, accessible and personalized therapy experience. However, these benefits must be balanced with the risks associated with over-reliance on technology, ethical concerns around data privacy, clinical implications for the therapeutic relationship, and challenges specific to art therapy practice, such as potentially diminished sense of ownership over (co)created artwork. Creative AI, whose value is yet to be examined, could potentially become the next addition to the art therapy toolkit. If managed carefully, it can add new dimensions to the therapeutic process and relationship, opening opportunities for novel ways of working therapeutically. As our knowledge of the application of conversational and embodied AI to psychotherapy grows, art therapy practice is where creative AI is by its nature most relevant and potentially transformative.

Art therapists are undisputedly central to shaping further developments in AI use in arts-based therapeutic interventions and ensuring that their values, methods and impacts are aligned with the discipline’s code of practice. Considering the current lack of more specific guidance, we hope that the art therapy community remain open minded about the yet unexplored opportunities of creative AI, while protecting human-centeredness, empathy and authentic creativity that are foundational to the art therapy process and unlikely to be challenged by AI anytime soon.

## Data Availability

The original contributions presented in the study are included in the article/supplementary material, further inquiries can be directed to the corresponding author.
